# Lifestyle Patterns Are Associated with Elevated Blood Pressure among Qatari Women of Reproductive Age: A Cross-Sectional National Study

**DOI:** 10.3390/nu7095355

**Published:** 2015-09-09

**Authors:** Mohammed Al Thani, Al Anoud Al Thani, Walaa Al-Chetachi, Badria Al Malki, Shamseldin A. H. Khalifa, Ahmad Haj Bakri, Nahla Hwalla, Lara Nasreddine, Farah Naja

**Affiliations:** 1 Public Health Department, Supreme Council of Health, Doha, Al Rumaila West, 42 Doha, Qatar; E-Mail: malthani@sch.gov.qa; 2 Health Promotion and Non Communicable Disease Prevention Division, Supreme Council of Health, Doha, Al Rumaila West, 42 Doha, Qatar; E-Mails: aalthani@sch.gov.qa (A.A.A.T.); walchetachi@sch.gov.qa (W.A.-C.); balmalki@sch.gov.qa (B.A.M.); skhalifa1@sch.gov.qa (S.A.H.K.); abakri@sch.gov.qa (A.H.B.); 3 Nutrition and Food Sciences Department, Faculty of Agriculture and Food Sciences, American University of Beirut, P. O. Box 11-0.236 Riad El Solh, 11072020 Beirut, Lebanon; E-Mail: nahla@aub.edu.lb

**Keywords:** lifestyle pattern, elevated blood pressure, factor analysis, women, Qatar

## Abstract

Women of childbearing age are particularly vulnerable to the adverse effects of elevated blood pressure (BP), with dietary and lifestyle habits being increasingly recognized as important modifiable environmental risk factors for this condition. Using data from the National STEPwise survey conducted in Qatar in year 2012, we aimed to examine lifestyle patterns and their association with elevated BP among Qatari women of childbearing age (18–45 years). Socio-demographic, lifestyle, dietary, anthropometric and BP data were used (*n* = 747). Principal component factor analysis was applied to identify the patterns using the frequency of consumption of 13 foods/food groups, physical activity level, and smoking status. Multivariate logistic regression analyses were used to evaluate the association of the identified lifestyle patterns with elevated BP and to examine the socio-demographic correlates of these patterns. Three lifestyle patterns were identified: a “healthy” pattern characterized by intake of fruits, natural juices, and vegetables; a “fast food & smoking” pattern characterized by fast foods, sweetened beverages, and sweets, in addition to smoking; and a “traditional sedentary” pattern which consisted of refined grains, dairy products, and meat in addition to low physical activity. The fast food & smoking and the traditional & sedentary patterns were associated with an approximately 2-fold increase in the risk of elevated BP in the study population. The findings of this study highlight the synergistic effect that diet, smoking and physical inactivity may have on the risk of elevated BP among Qatari women.

## 1. Introduction

Globally, hypertension is the third leading cause of mortality and is a major risk factor for heart disease, stroke, and kidney failure [1,2]. According to the World Health Organization (WHO), 22.2% of the adult population in 2014 had hypertension (24% of men and 20.5% of women) [3]. This prevalence was projected to increase to 29.2% (29% of men and 29.5% of women) by year 2025 [4]. Among countries contributing significantly to this alarming increase in hypertension prevalence are countries of the Gulf Cooperation Council, where fast economic growth was accompanied by a steep rise in nutritional health problems and related disease. Qatar has recently emerged as the richest country in the world in terms of Gross Domestic Product (GDP) per capita. Though milestones have been accomplished in the betterment of life expectancy and health in the country, the WHO World Health Survey (WHS) in 2006 and later the WHO STEPwise survey in 2012 identified lifestyle-related non-communicable diseases, including obesity, diabetes and hypertension, as main health challenges, with hypertension rates increasing by 75% during this time interval [5,6].

Of all adults, women of childbearing age are particularly vulnerable to the adverse effects of elevated blood pressure (BP) and hypertension. In addition to its effect on morbidity and mortality on the mother, hypertension in women of child-bearing age has been shown to be a major risk factor during pregnancy for both mother and child, possibly due to changes in blood flow to the uterus during pregnancy [7]. One in four women with pre-existing hypertension experience superimposed preeclampsia during pregnancy, placing the mother at high risk of organ damage and often necessitating early childbirth [8]. Furthermore, rates of preterm delivery, low birth weight, neonatal unit admission, and perinatal death are around three to four times higher among mothers with hypertension [7]. A recent systematic review and meta-analysis showed significant associations between maternal hypertension and congenital heart disease risk in the offspring (Relative Risk (RR) 1.8; 95% Confidence Interval (CI) 1.5, 2.2) regardless of whether hypertension was treated or not [9]. These substantial short and long-term health risks of hypertension among women of child-bearing age make this age group an ideal target for the primary prevention of hypertension.

The escalating trend in hypertension prevalence coupled to significant downstream pathophysiological effects and enormous financial liabilities pose major public health concerns that necessitate the search for mitigating factors and strategies to address them. Prehypertension and elevated BP are recognized as frequent precursors of hypertension [10]. Dietary intake is an important modifiable environmental risk factor in the development and prevention of elevated BP and hypertension [11]. Studies on the association between dietary factors and BP have consistently shown that excess sodium and alcohol intake and inadequate intake of potassium increase BP [12]. However, the evidence for the association with other dietary factors has been less consistent. For instance, dietary protein appeared to reduce BP in a few randomized trials; however, such a relationship was not confirmed by long-term studies [13]. Furthermore, the optimal source of protein (e.g., plant or animal) implicated in BP reduction has not been also identified. It is also not clear whether the reduction of BP with a higher intake of protein is rather due to a concomitant reduction in carbohydrates [13]. Similarly, while high fiber intake has shown a modest reduction on BP [14], the benefits of dietary fiber are difficult to distinguish from the benefits of an increase in vitamins and minerals, such as potassium, coming from the same fibrous plant-based foods [12]. To overcome such inconsistencies, nutrition epidemiologists have proposed studying dietary patterns as an alternative approach to single nutrients in evaluating diet-disease relationships [15]. Several studies have investigated the association between dietary patterns and hypertension [12,16,17], consistently showing an increased risk for hypertension with higher adherence to a “Western” dietary pattern [17].

Though dietary intake is a main modulator of BP and risk of hypertension, other behavioral factors such as smoking and physical activity have also been implicated as risk factors of hypertension and are important to consider in addressing prevention. Studying a single behavioral risk factor overlooks the fact that people are exposed to a combination of risk factors which are frequently interactive and/or synergistic [18]. Recently, a few studies have addressed the association between lifestyle patterns and weight status among children [19]; dysglycemia [20] and non-communicable diseases [18]. Not only does this approach account for the collinearity or intercorrelations between risk factors, it allows for a better understanding of high-risk behaviours as they cluster in real-life and thus produces more culturally sensitive public health recommendation that can be easily interpreted and followed by the general population.

Using data from the National WHO STEPwise s survey conducted in Qatar in year 2012, this study aimed at identifying lifestyle patterns among women of reproductive age in Qatar and investigating the association of these patterns with elevated BP. A secondary objective was to examine the socio-demographic correlates of the identified lifestyle patterns. Findings of this study will pave the way for the development of culturally sensitive evidence-based interventions strategies aimed at preventing elevated BP among women of child-bearing age in Qatar.

## 2. Methodology

### 2.1. Study Design

The data presented in this paper is a secondary analysis of the National STEPwise Survey conducted in Qatar during year 2012. The survey was conducted on a nationally representative sample of Qatari adults aged between 18 and 64 years. The survey design, including sampling and data collection, was modelled based on the WHO STEPwise approach to non-communicable disease risk factor surveillance. The sample consisted of randomly selected households based on a multi-stage cluster sampling. Clusters were selected from the seven municipalities of Qatar (Doha, Al Rayyan, Al Warka, Umm Salal, Al Khor, Al Shamal, Al Daayeen). The clusters were defined as a group of contiguous blocks (between 60 and 70 blocks). Using probability proportional to size sampling, a systematic random sample of 95 clusters was selected [21]. Within each cluster, 30 households were randomly chosen. Adults of Qatari nationality between the age of 18 and 64 years were eligible to participate in the survey. In the household, in case more than one subject was eligible, using a personal digital assistant (PDA) to generate a random number, only one adult was randomly selected. Out of the 2850 households approached, 2496 participated in the study (response rate 88%). The Qatar WHO STEPwise survey protocol was granted ethical approval from the Supreme Council of Health and the Ministry of Development Planning and Statistics, Doha, Qatar. A written consent form was obtained from all study participants who were assured that all information they provided was strictly confidential, their participation is voluntary, and they have the right to refuse the participation and withdraw from the interview at any stage. Further details about the design and protocol of the Qatar STEPwise survey are found at Haj Bakri & Al-Thani (2013) [6]. The present paper focused on women of child bearing age. The selection criteria were (1) female sex; (2) age between 18 and 45; (3) healthy (no known diagnosis of hypertension, other chronic diseases, or conditions that may affect dietary intake); and (4) not pregnant. Out of 776 women who meet the inclusion criteria, 29 had missing or incomplete data for BP, dietary and/or lifestyle information and hence were excluded from the analysis.

### 2.2. Data Collection

The participants were visited at their household by interviewers who were trained on the methodology of the survey and protocols of data collection prior to the initiation of field work. During the interview, participants responded to the questions of the interviewers which were based on a standard multi-component questionnaire and they have also undergone physical examination. The questionnaire included information about socio-demographic (age, sex, education, marital status, job type, parental consanguinity, family history of diabetes and hypertension) and lifestyle characteristics (smoking, physical activity and dietary intake). Smoking questions addressed the smoking status of the participants as well as his/her exposure to passive smoking (days/week). As recommended by the WHO STEPwise approach, the Arabic Global Physical Activity Questionnaire (GPAQ) was used to assess physical activity. The GPAQ covers several components of physical activity, such as intensity, duration, and frequency, and it assesses three domains in which physical activity is performed (occupational physical activity, transport-related physical activity, and physical activity during discretionary or leisure time) [22]. Total physical activity was calculated by weighting each type of activity by its energy requirements defined in MET (Metabolic Equivalent of Task)-minutes. The total MET-min were computed as the sum of all MET-min/week from moderate-to-vigorous-intensity physical activities performed for each of the three domains, and was later converted to total Met-min per day [23]. Three categories of physical activity (low, moderate, high) were assigned based on METS-min per week [24]. Dietary intake was assessed using a non-quantitative (without reference to portion size) food frequency questionnaire. A total of 13 food groups were included: refined grains, fruits, vegetables, milk and dairy products, meat, poultry, fish and sea food, beans, sweets, sweetened beverages, whole grains, and natural juices. Frequency of consumption was recorded as number of days per week the food/food group was consumed. No questions on alcohol were included for cultural and religious considerations. Besides providing information on usual intakes of a particular food or food groups of interest, such non-quantitative Food Frequecy Questionnaires (FFQs) have been particularly useful in identifying dietary patterns at the population level [25]. All components of the questionnaire were tested for cultural sensitivity on a sample of Qatari adults prior to the initiation of field work.

During physical examination, the BP, weight, height and waist circumference of participants were determined. BP, both systolic and diastolic, was measured through a calibrated Omron M7 sphygmomanometer (Omron BP785; China). Three readings were obtained for both systolic and diastolic BP at 5 minutes intervals. The average of the second and the third readings were used. Elevated BP was defined as either systolic pressure ≥ 130 or diastolic pressure ≥ 85 mm Hg [26,27].

Weight and height measurements were taken using standardized techniques and calibrated equipment. Subjects were weighed to the nearest 0.1 kg in light indoor clothing and with bare feet or stockings. Using a stadiometer, height was measured without shoes and recorded to the nearest 0.5 cm. Body mass index (BMI) was calculated as the ratio of weight (kilograms) to the square of height (meters).

### 2.3. Lifestyle Patterns Derivation

Using the exploratory Principal Component Factor Analysis (PCFA), lifestyle patterns were identified based on the frequency of consumption of the 13 foods/food groups, physical activity (in Mets-Minutes), and smoking status (non-smoker, past smoker and current smoker). Prior to running the PCFA, the correlation matrix between all the variables was visually and statistically examined to justify undertaking the analysis. The chi square for Bartlett test of sphericity was significant at a *p*-value less than 0.05, and the Kaiser-Meyer-Olkin test (KMO) was greater than 0.6, indicating that the correlation among the variables was sufficiently strong for a factor analysis. The number of factors retained was based on three criteria: (1) the Kaiser criterion (eigenvalues > 1); (2) inflection point of the scree plot (3) and the interpretability of factors. The factors were rotated by a Varimax rotation (orthogonal transformation). Factor loadings indicated the strength and direction of the association between the patterns and the lifestyle variables. Factor scores were calculated by multiple regression approach with each participant possessing a score on each of the three factors. These scores indicated the degree to which each subject’s diet adheres to the identified pattern. For each pattern, participants were grouped into tertiles of pattern scores.

### 2.4. Statistical Analyses

In order to correct for the selection probabilities, the sample distribution was calibrated to the Qatari population totals using geographical and 5 year-age distributions. Data analysis was weighted using sampling weights calculated as the inverse of the sampling fractions. In weighting, the distribution of the 2010 Qatari population by municipality and 5 year age group was used as the reference [21]. Socio-demographic, lifestyle characteristics, eating habits and anthropometric measurements were described using means ± standard deviations (SD) and proportions for continuous and categorical variables, respectively. The chi-square test, *t*-test and ANOVA were used to chart comparisons between groups. Multiple logistic regression analyses were applied to identify the socio-demographic and lifestyle correlates of adherence to the identified patterns. Adherence was defined as belonging to the third tertile of the pattern score. The associations of the lifestyle patterns with elevated BP were evaluated by means of multivariate logistic regression models, with tertiles of dietary patterns’ scores as independent variables and elevated BP as the outcome variables. Belonging to the third tertile of a certain pattern’s score indicated that the participants had higher adherence to this pattern when compared to other participants who belonged to the first and second tertiles. The multivariate logistic regression models were adjusted for variables found to be associated with either BP or the lifestyle patterns and these included age, education, marital status, parental consanguinity, family history BP, number of meals not eaten at home, exposure to passive smoking and BMI. The Statistical Package for the Social Sciences (SPSS, version 14.1, Chicago, IL, USA) was used for all computations [28] and a *p*-value < 0.05 was considered significant.

## 3. Results

Socio-demographics, lifestyle and anthropometric characteristics of study participants by BP status are presented in Table 1. Out of the 747 women study participants, 105 (14%) had an elevated BP. Study participants’ mean age was 31.0 ± 7.0 years. Eighty % of subjects had high school or higher education level with almost 52% working (either government (47.7%) or private (4%) employment), 29.6% were housewives. A considerable proportion (35.1%) reported parental consanguinity. Family history of diabetes and hypertension were reported among 68% and 64.3% of participants, respectively. The majority of subjects were non-smokers (97.7%) with only 1.5% being current smokers. Over 50% of subjects belonged to the low physical activity category, with walking constituting 55.5% of total physical activity. This percentage was higher than work (26.2%) and leisure time (18.3%) related activities. Thirty eight percent of subjects were obese (BMI ≥ 30 kg/m^2^). Comparisons between subjects with elevated *versus* those with normal BP showed that subjects with elevated BP were older (33.5 ± 6.9 *vs.* 30.5 ± 6.9, *p* < 0.01), more likely to be married (73.6% *vs.* 63.7%; *p* < 0.05); and have a family history of BP (77.4% *vs.* 62.1%; *p* < 0.05). Furthermore, obesity was more prevalent among subjects with elevated BP compared to those with normal BP (50.0% *vs.* 35.2%, *p* < 0.05).

**Table 1:** Weighted socio-demographics, lifestyle and anthropometric characteristics of study participants by blood pressure status ^a^ (*n* = 747).

Variable Name	Total *n* = 747	Normal Blood Pressure *n* = 642	Elevated Blood ^†^ Pressure *n* = 105	Significance ^††^
**Age (years)**	31.0 ± 7.0	30.5 ± 6.9	33.5 ± 6.9	*p* = 0.000 **
**Education**				
Up to intermediate level ^b^	140 (20)	113 (17.6)	24 (22.9)	*p* = 0.36
Finished high school	280 (40)	248 (38.7)	35 (33.3)	
University/graduate level	326 (43.7)	280 (43.7)	46 (43.8)	
**Marital Status**				
Not married	261 (34.9)	233 (36.3)	28 (26.4)	*p* = 0.05 *
Married	486 (65.1)	409 (63.7)	78 (73.6)	
**Job type**				
Governmental employee	356 (47.7)	309 (48.1)	47 (44.8)	*p* = 0.42
Non-governmental employee ^c^	30 (4.0)	26 (4.0)	4 (3.8)	
Not working	140 (18.7)	124 (19.3)	16 (15.2)	
Housewife	221 (29.6)	183 (28.5)	38 (36.2)	
**Parental Consanguinity**				
No	485 (64.9)	417 (65.0)	68 (64.8)	*p* = 0.97
Yes	262 (35.1)	225 (35.0)	37 (35.2)	
**Family history of diabetes**				
No	240 (32.1)	207 (32.2)	33 (31.4)	*p* = 0.87
Yes	507 (67.9)	435 (67.8)	72 (68.6)	
**Family history of High blood pressure**				
No	267 (35.7)	243 (37.9)	24 (22.6)	*p* = 0.002 *
Yes	480 (64.3)	399 (62.1)	82 (77.4)	
**Oil type used in cooking**				
Vegetable oil	714 (96.4)	613 (96.2)	101 (97.1)	*p* = 0.66
Animal oil	27 (3.6)	24 (3.8)	3 (2.9)	
**Number of Meals not prepared at home (per week)**	2.4 ± 2.3	2.5 ± 2.4	2.1 ± 2.0	*p* = 0.10
**Smoking Status**				
Non smoker	730 (97.7)	628 (97.8)	102 (97.1)	*p* = 0.28
Past smoker	6 (0.8)	6 (0.9)	0 (0.0)	
Current smoker	11 (1.5)	8 (1.2)	3 (2.9)	
**Exposure to passive Smoking ^d^ (days/week)**	1.2 ± 2.9	1.2 ± 2.9	1.3 ± 3.0	*p* = 0.71
**Physical Activity Level**				
Low	416 (55.8)	352 (54.9)	64 (61.0)	*p* = 0.41
Moderate	162 (21.7)	144 (22.5)	18 (17.1)	
High	168 (22.5)	145 (22.6)	23 (21.9)	
**Total physical activity (Met-minutes per day)**	389.9 ± 761.9	394.5 ± 772.7	369.2 ± 701.5	*p* = 0.75
**Percent activity from work (%) (*n* = 551)**	26.2 ± 37.7	25.5 ± 37.2	31.7 ± 40.6	*p* = 0.22
**Percent activity from walking (%)**	55.5 ± 40.8	55.6 ± 41.1	54.3 ± 39.8	*p* = 0.80
**Percent activity form free time (%)**	18.3 ± 30.8	18.9 ± 31.7	14.1 ± 23.9	*p* = 0.15
**Sedentary time (minutes/day)**	183.6 ± 168.3	183.0 ± 164.4	189.5 ± 191.2	*p* = 0.71
**Body mass index(BMI) (kg/m^2^)**	29.1 ± 7.2	28.7 ± 7.1	30.9 ± 6.9	*p* = 0.004 *
Obese(≥30 kg/m^2^)	279 (37.3)	226 (35.2)	53 (50.0)	*p* = 0.007 *

**^†^** Elevated blood pressure in this study was defined as either systolic pressure ≥130 or diastolic pressure ≥85 mm Hg; **^††^** *p*-values were derived from *t* test and Chi Square test for continuous and categorical variables respectively; ^a^ Percentages are within column; ^b^ This category includes: no schooling, elementary and intermediate schooling; ^c^ This category includes: private and own business; ^d^ This includes passive smoking from family members and at work; * *p* ≤ 0.05; ** *p* ≤ 0.001.

**Figure 1:**
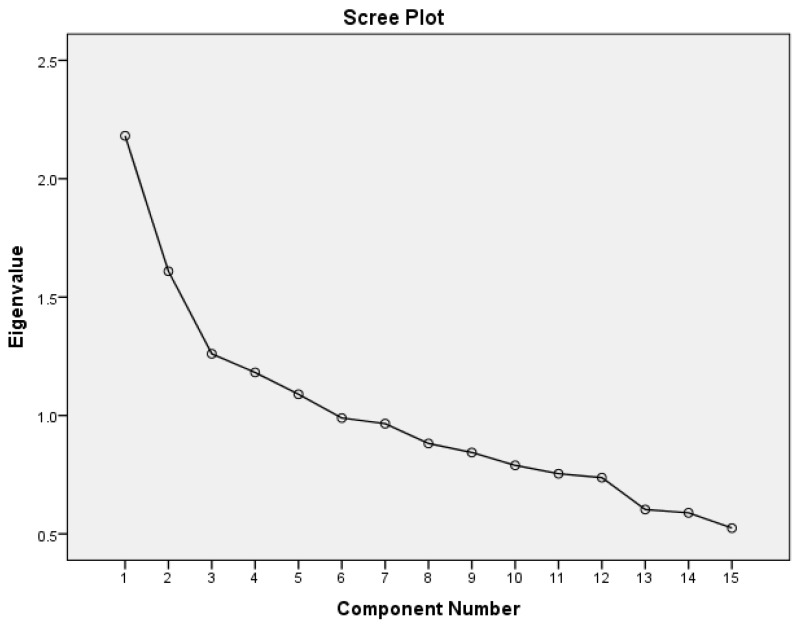
Scree plot showing eigenvalues for the 15 components, extracted in weighted factor analysis of lifestyle and dietary data of study participants (*n* = 747).

Figure 1 is the scree plot showing eigenvalues for the 15 components derived from the lifestyle and dietary data using PCFA. Examination of the scree plot revealed a clear inflection at the third component (Figure 1). Taken together with the Kaiser criterion of an eigenvalue >1, it was deduced that three components/factors ought to be retained.

Factor loadings and the variance explained by each of the retained three patterns are shown in Table 2 (loadings greater than 0.3 are bolded). Together these patterns explained 34.1% of the variance (“Healthy”: 12.4%; “Fast food & smoking”: 12.1% and “Traditional sedentary”: 9.6%). The “Healthy” pattern wascharacterized by a high intake of fruits, beans, natural juices, vegetables, fish, and whole grains. In addition to smoking, the “Fast food & smoking” pattern was characterized by fast foods, sweetened beverages, sweets and poultry. The “Traditional sedentary” pattern consisted mainly of refined grains, meat and low physical activity. It is important to note that beans loaded on the “Traditional sedentary” pattern (0.26) in addition to the “Fast food & smoking” pattern (0.56). Whole grains had a high negative loading on the “Traditional sedentary” pattern (−0.55).

**Table 2:** Weighted factor loading matrix of the three identified lifestyle patterns among a nationally representative sample of Qatari women ^a^ (*n* = 747).

	Lifestyle Patterns		
	Healthy	Fast Food & Smoking	
Fruits	**0.68**	−0.20	
Beans	**0.56**		0.27
Natural juices	**0.55**		
Vegetables	**0.44**		
Fish	**0.44**		−0.21
Dairy	0.24		0.21
Fast foods		**0.78**	
Sweetened beverages		**0.63**	
Sweets		**0.54**	
Smoking		**0.39**	
Poultry		**0.34**	
Refined grains		0.22	**0.74**
Whole grains	**0.32**	−0.21	**−0.55**
Physical activity (Mets/day)			**−0.43**
Meat			**0.30**
Percent variance explained	12.4	12.1	9.6

^a^ Factor loadings of less than |0.2| were not listed in the table for simplicity. Loadings ≥ 0.3 are bolded.

To further characterize the identified patterns, the dietary intakes, physical activity, smoking characteristics of study participants were described for the first and third tertiles of each of the patterns’ scores (Table 3). Compared to participants in the first tertile of the “Healthy” pattern, those belonging to the third tertile had significantly higher frequencies (days/week) of consumption of fruits, beans, natural juices, vegetables, fish and sea food, dairy products, and whole grains, and had lower frequencies of consumption of sweetened beverages and poultry. As for the “Fast food & smoking” pattern, frequencies of consumption of beans, fast food, sweetened beverages, sweets, poultry, refined grains, and meat were higher among subjects in the third tertile compared to those in the first tertile. Fruits, vegetables and whole grains frequencies of consumption were lower among subjects in the third compared to the first tertile of this pattern. Furthermore, a higher proportion of smokers was found among subjects in the third tertile of the “Fast food & smoking” pattern. Foods/food groups with higher frequency of consumption among subjects in the third as compared to the first tertile of the “Traditional sedentary” pattern were beans, dairy products, sweetened beverages, sweets, poultry, refined grains, and meat. In contrast, lower frequency of consumption was noted for fruits, natural juices, vegetables, fish and sea food, fast food and whole grains. Though no difference in physical activity was noted for any of the first two patterns, subjects belonging to third tertile of the “Traditional sedentary” pattern had significantly lower levels of physical activity compared to those belonging to the first tertile (Table 3).

Table 4 displays the association of the derived lifestyle patterns with elevated BP as evaluated by multivariate logistic regression analyses. After adjustment for socio-demographic characteristics and BMI, the results showed that subjects belonging to the second tertile of the “Fast food & smoking” pattern had a two-fold increase in the odds of elevated BP compared to those in the first tertile (Odds Ratio (OR): 2.1, 95% CI: 1.4–3.3). A similar association was observed for subjects in the third compared to the first tertile of this pattern however the odds did not reach significance. The small number of smokers in the study sample may not have provided enough power to detect a significant association in the third tertile. Furthermore, a gradual increase in the odds of elevated BP was observed with a higher adherence to the “Traditional sedentary” pattern reaching OR: 2.2, 95% CI 1.3–3.7 for the third compared to the first tertile of this pattern (*p* for trend <0.05) (Table 4).

**Table 3:** Weighted dietary intake, smoking, and physical activity of study participants by tertiles of the three identified patterns’ scores ^a^ (*n* = 747).

Factor Items	Healthy		Fast Food & Smoking		Traditional Sedentary	
	1st Tertile	3rd Tertile	1st Tertile	3rd Tertile	1st Tertile	3rd Tertile
Mean ± SD						
**Fruits (days/week)**	1.2 ± 1.4	5.1 ± 2.4 **	3.6 ± 2.7	2.3 ± 2.3 **	3.5 ± 2.7	2.7 ± 2.3 *
**Beans (days/week)**	0.8 ± 0.9	2.7 ± 2.1 **	1.3 ± 1.4	2.0 ± 2.1 **	1.2 ± 1.4	2.1 ± 2.0 **
**Natural juice(days/week)**	1.9 ± 2.0	5.3 ± 2.3 **	3.4 ± 2.7	3.5 ± 2.7	3.8 ± 2.8	3.0 ± 2.5 *
**Vegetables (days/week)**	4.0 ± 2.7	6.5 ± 1.4 **	5.7 ± 2.2	5.0 ± 2.5 *	5.5 ± 2.3	5.4 ± 2.3
**Fish and sea food (days/week)**	0.8 ± 0.8	2.2 ± 1.6 **	1.6 ± 1.3	1.3 ± 1.3 *	1.7 ± 1.5	1.1 ± 1.1 **
**Dairy products (days/week)**	5.4 ± 2.5	6.2 ± 1.8 **	5.9 ± 2.1	5.9 ± 2.2	5.5 ± 2.4	6.3 ± 1.7 **
**Fast food (days/week)**	1.7 ± 1.9	1.9 ± 2.0	0.6 ± 0.8	3.6 ± 2.3 **	1.9 ± 2.2	1.3 ± 1.4 **
**Sweetened beverages(days/week)**	2.9 ± 2.9	2.2 ± 2.7 *	0.6 ± 1.2	5.1 ± 2.6 **	2.2 ± 2.7	2.4 ± 2.7 *
**Sweets (days/week)**	4.3 ± 2.8	4.2 ± 2.6	2.4 ± 2.3	5.9 ± 1.9 **	3.9 ± 2.7	4.7 ± 2.6 *
**Smoking Status ^b^**						
Non smoker	239 (97.2)	229 (99.1)	259 (100.0)	205 (94.0) **	233 (94.7)	248 (100.0) **
Past smoker	1 (0.4)	2 (0.9)	0 (0.0)	10 (4.6)	3 (1.2)	0 (0)
Current smoker	6 (2.4)	0 (0.0)	0 (0.0)	3 (1.4)	10 (4.1)	0 (0)
**Poultry(days/week)**	5.4 ± 2.2	4.7 ± 2.1 *	4.0 ± 2.3	5.9 ± 1.8 **	4.4 ± 2.3	5.6 ± 2.0 **
**Refined grains (days/week)**	5.6 ± 2.4	5.2 ± 2.5	4.7 ± 2.8	5.8 ± 2.1 **	2.9 ± 2.5	7.0 ± 0.2 **
**Whole grains (days/week)**	0.9 ± 1.9	2.8 ± 2.9 **	2.6 ± 2.9	1.4 ± 2.3 **	4.0 ± 3.0	0.4 ± 1.1 **
**Total physical activity (Met-minutes per day)**	450 ± 770	372.3 ± 672.9	386.0 ± 677.9	463.5 ± 844.9	627.5 ± 1054.0	203.4 ± 429.0 **
**Meat (days/week)**	1.3 ± 1.4	2.0 ± 1.6 **	1.4 ± 1.3	1.9 ± 1.8 *	1.2 ± 1.4	2.1 ± 1.8 **

^a^ Intake of the various food groups referred to frequency of consumption as expressed by number of days per week the food/food group was consumed; ^b^ For smoking the numbers represent *n* (%); * *p* ≤ 0.05; ** *p* ≤ 0.001.

**Table 4:** Weighted odds ratio and their 95% Confidence Interval (CI) for the association of the identified lifestyle patterns with elevated blood pressure in the study population (*n* = 747).

	Dietary Patterns		
	Healthy	Fast Food & Smoking	Traditional Sedentary
Age adjusted model			
1st tertile	Ref.	Ref.	Ref.
2nd tertile	1.3 (0.8–2.1)	**2.1 (1.3–3.2)**	2.1 (1.2–3.5)
3rd tertile	1.6 (0.9–2.5)	1.2 (0.7–2.0)	**2.2 (1.3–3.7)**
Multivariate model 2 ^a^			
1st tertile	Ref.	Ref.	Ref.
2nd tertile	1.3 (0.8–2.2)	**2.1 (1.4–3.3)**	**2.0 (1.2–3.5)**
3rd tertile	1.4 (0.9–2.2)	1.1 (0.7–2.0)	**2.2 (1.3–3.7)**

^a^ This model is adjusted for age, education, marital status, parental consanguinity, family history of blood pressure, number of meals not eaten at home, exposure to passive smoking and Body mass index (BMI).

Multivariate logistic regression models were used in order to determine the correlates of each of the lifestyle patterns. In each of the models, adherence to the lifestyle pattern (belonging to the third tertile *vs.* second and first tertiles) was the outcome with all socio-demographic and lifestyle characteristics as independent variables. Results showed that a higher education level was associated with the “Healthy” pattern (OR: 2.0, 95% CI: 1.2–3.2). Adherence to the “Fast food & smoking” pattern was associated with a consumption of a higher number of meals outside home (OR: 1.5, 95% CI: 1.4–1.7) and a higher exposure to passive smoking (OR: 1.10, 95% CI: 1.04–1.16). Furthermore, older subjects were less likely to adhere to this pattern (OR: 0.97, 95% CI: 0.94–0.98). While parental consanguinity was associated with a greater adherence to the “Traditional sedentary” pattern in the study sample (OR: 1.4, 95% CI: 1.0–1.9), a higher level of education was associated with a lower adherence to this pattern (OR: 0.6, 95% CI: 0.4–1.0).

## 4. Discussion

To our knowledge, this is the first study to investigate lifestyle patterns and their association with elevated BP. Using data stemming from the National STEPwise survey conducted in Qatar in year 2012, we identified three lifestyle patterns amongst Qatari women of reproductive age, with only the “Fast food & smoking” and “Traditional sedentary” patterns being associated with increased risk of raised BP. Together, the identified patterns explained 33.7% of the variance, which falls within the range reported in the literature (23.5%–45%) [18,20,29]. Only a few studies have adopted the lifestyle pattern approach and aimed at investigating the combined effects of food intake and other health-related lifestyle characteristics on disease risk [18,20,29]. A brief description of these studies is presented in the table below (Table 5).

**Table 5:** Summary of studies investigating lifestyle factors and their associations with disease among adults.

Authors’s Name	Study Population	Disease Outcome	Lifestyle Factors	Main Findings
Navarro Silvera *et al.* (2011) [29] *USA*	*n*: 1782 Age: 30–79 years	Subtypes of Esophageal and Gastric Cancer ^a^	Meat & nitrate Fruit & vegetable Smoking & alcohol Legume & meat alternate Gastroesophageal reflux disease (GERD) & body mass index (BMI) Fish & vitamin C	“Meat & nitrate” intake associated with increased risk of EA, GCA, and OGA “Fruit & vegetable” associated with reduced risk of EA, ESCC, and GCA “Smoking & alcohol” associated with increased risk of ESCC “GERD & obesity” associated with increased risk of EA and ESCC “Fish & vitamin C” associated with increased risk of ESCC
Steele *et al.* (2014) [18] *Brazil*	*n*: 108,706 Age: ≥18 years Sex: 61.3% female	N/A	Prudent pattern: regular consumption of fruit and vegetables, daily fresh-fruit juice, and fat-reduced milk; physical activity practice, protection against UV radiation, reduced soft drink consumption Risky pattern: fat-rich meat consumption, excessive alcoholic beverage intake, current smoking, excess TV watching (especially in men), regular soft drink consumption (especially in women)	N/A
Waidyatilaka *et al.* (2014) [20] *Sri Lanka*	*n*: 617 Age: 30–45 years Sex: 100% females	Cardiometabolic risk variables ^b^	Pattern 1: rice and rice flour-based products, pulses, seafood, fruits, vegetables and green leafy vegetables Pattern 2: wheat, wheat based products and tubers, red meat, and processed meat Pattern 3: snacks dairy products and poultry, low physical activity	Pattern 1 has no association with dysglycaemic risk Patterns 2 and 3 positively associated with dysglycaemic risk. Pattern 1 associated with increased HDL and reduced TC and TAG Pattern 2 associated with increased WC, BMI, FM% and hs-CRP and reduced FFM% and HDL Pattern 3 associated with increased WC, BMI, FM%, HbA1c, FBS, TC, TAG, and hs-CRP and reduced FFM% and HDL

^a^ Subtypes of Esophageal and Gastric Cancer: Esophageal adenocarcinoma (EA), esophageal squamous cell carcinoma (ESCC), gastric cardia adenocarcinoma (GCA), other gastric cancers (OGA); ^b^ Cardiometabolic risk variables: dysglycaemic risk, waist circumference (WC), fat mass percentage (FM%), fat-free mass percentage (FFM%), glycosylated Hb (HbA1c), fasting blood sugar (FBS), total cholesterol (TC), high sensitivity C-reactive protein (hs-CRP), High Density Lipoproteins (HDL); TriAcylGlycerids (TAG).

As not only diet but also other lifestyle characteristics such as smoking and physical activity are known to increase the risk for elevated BP and other non-communicable diseases (NCDs), the measurement of the combined effect of these variables on disease risk provides valuable information for evidence-based and culturally sensitive primary prevention and health promotion. It was in fact suggested that disease risk increases with the number of unhealthy behaviors such as smoking, unhealthy diet and sedentary behavior [18] and that some of these unhealthy behaviors may interact to produce an even greater risk than if the individual risks are added together [30].

The findings of this study showed that the “Healthy” pattern, characterized by the consumption of plant-based foods (fruits, natural juices, vegetables, beans, whole grains) and fish, was not associated with increased risk of elevated BP in the study population. The “Healthy” pattern of this study shares many characteristics of what is usually described in the literature, as the “Prudent” or “Healthy” dietary pattern [17]. Some of the studies investigating dietary patterns in relation to the risk of hypertension have reported a protective effect from the “Prudent” or “Healthy” pattern [31,32], while others, and in accordance with our findings, have reported a lack of association [33,34,35]. Available evidence suggests that plant-based dietary patterns, including diets rich in fruits, vegetables, and combination diets such as the DASH (Dietary Approaches to Stop Hypertension), may be associated with BP reductions in both hypertensive and normotensive individuals [36,37]. Plant-based foods are in fact good sources of potassium, magnesium, dietary fiber and anthocyanins, all of which have been suggested to exert beneficial effects on BP regulation [12]. The “Healthy” pattern was also characterized by a higher frequency of fish consumption, which may imply a higher intake of omega 3 fatty acids. Available evidence suggests that high intakes of fish oils from supplements may be associated with BP reduction, while evidence on naturally occurring omega 3 fatty acids from fatty fish is less convincing [12]. This highlights, once more, the importance of adopting a holistic pattern dietary analysis approach, rather than investigating the consumption of individual foods, when looking at factors associated with disease risk [12]. In this context, it is important to note that the fact that the “Healthy” pattern did not load on physical activity may have diluted the protective effects of its dietary components, thus explaining the lack of association between the “Healthy” pattern and BP. In fact, substantial evidence links higher levels of physical to lower risks of hypertension and underlines a dose-dependent inverse relationship between levels of physical activity and BP [11]. The recent guidelines of the American College of Cardiology and the American Heart Association for the reduction of cardiovascular risk, including elevated BP, stress a combination of lifestyle intervention strategies that incorporate higher physical activity levels with healthy dietary patterns emphasizing vegetables, fruits, whole grains, fish, and legumes [11].

The “Fast food & smoking” pattern, which is characterized by smoking and the consumption of fast foods, sweetened beverages, and sweets, was associated with a two-fold increase in the risk of elevated BP amongst Qatari women of reproductive age, even after adjustment for potential confounders including BMI. As such, our findings demonstrate the synergistic effects of smoking with dietary habits on disease risk. Available evidence suggests that smoking is associated with increased BP, even though a clear causal relationship has not yet been documented. In a prospective cohort study based on the Women’s Health Study, smoking was found to be associated with an increased risk of developing hypertension, with the strongest effect being documented among women smoking at least 15 cigarettes per day [38]. The increase in BP in smokers may depend on several factors including (1) the toxic effects of carbon monoxide and other smoking-related chemical compounds on the arterial wall; (2) the increase in red blood cell number and blood viscosity-secondary to carbon monoxide exposure [39] and (3) sympathetic and adrenergic stimulation, which is mainly caused by nicotine and its metabolites [40]. In addition to smoking, the “Fast food & smoking” pattern is also characterized by the consumption of fast food, which may imply a higher intake of salt, saturated fatty acids (SFA) and trans fatty acids (TFA). While high salt intake has been established as a risk factor for hypertension and elevated BP, the relationship between BP and the intakes of SFA and TFA is less conclusive [41,42,43]. It has been suggested that high SFA intakes may adversely affect vascular function and increase BP by proinflammatory mechanisms within the endothelium [43], while TFA consumption was associated with an impairment of endothelial function, as reflected by a reduction in brachial artery flow-mediated vasodilatation [44]. Higher frequency of consumption of sweets and sugar sweetened beverages (SSBs) was another characteristic of the “Fast food & smoking” pattern, thus implying a higher intake of sugar. Even though available evidence is conflicting, a recent systematic review and meta-analysis of randomized controlled trials showed that higher intakes of sugars are associated with increased BP levels [45]. High glucose intakes and postprandial hyperglycemia were suggested to impair vascular endothelial function by inducing lipid peroxidation and decreasing Nitric Oxide bioavailability [46]. High fructose intakes have been also mechanistically linked to an impairment of insulin signaling [47], increased lipogenesis [48] and disruption of vascular homeostasis [46,49]. Interestingly, it has been postulated that increased dietary fructose and salt may exert an additive effect on BP elevation [50]. Recent studies have in fact shed new light on the role of dietary fructose in enhancing salt absorption at the levels of the intestine and the kidney, highlighting a possible synergistic effect between fructose and salt in the development of hypertension [50]. Taken together, it can be noted that even though controversy characterizes the associations between individual dietary components and BP, our study shows that a lifestyle pattern in Qatar characterized by smoking coupled with dietary habits that may increase the intake of SFA, TFA, salt and sugar is associated with a two fold increase in elevated BP risk.

In this study, women who were predominantly physically inactive and consumed more meat and refined grains (“Traditional sedentary”) had also a significant increase in the risk of elevated BP. Physical inactivity has been repetitively identified as a risk factor for elevated BP and hypertension, with the protective effects of physical activity being documented in prehypertensive as well as hypertensive individuals [51,52,53]. A recent meta-analysis of 13 prospective cohort studies confirmed an inverse, dose–response association between levels of recreational physical activity and risk for developing hypertension [51,52]. Mechanistically, it was shown that physical activity significantly improves vascular function [54] and that habitual aerobic exercise training improves arterial stiffness [55] as well as endothelium-dependent vasorelaxation through increasing nitric oxide release [54]. The high prevalence of physical inactivity amongst Qatari women does not bode well for the future health profile of the population of Qatar. A higher frequency of red meat consumption was another characteristic of the “Traditional sedentary” pattern. The evidence on the association between meat and BP is controversial. In a prospective cohort of female US health professionals, red meat intake was positively associated with the risk of hypertension [56], while in a cross-sectional study on Dutch adults, meat protein was not found to be associated with incident hypertension [57]. In contrast, Ahhmed and Muguruma (2010) reported that meat protein may play a protective role against hypertension, a role that is mainly mediated by the angiotension-converting enzyme inhibitory activity of some of its protein hydorlysates [58]. A recent large prospective cohort of French women showed that while there was no association of unprocessed red meat consumption with hypertension, the consumption of processed red meat was significantly associated with a risk for hypertension [59]. Red meat in general, and processed meat in particular, are major sources of saturated fat and cholesterol, which have been suggested to have detrimental effects on BP control [56,60]. In addition, processed red meat is usually high in salt and contains various preservatives, additives, and other chemicals arising from food processing [56]. The advanced glycation and lipoxidation end products formed during the processing or cooking of red meat may impair insulin activity [61], induce inflammatory mediators [62] and may therefore impact BP regulation [56]. Qatar’s STEPwise survey did not differentiate between the intake of processed *vs.* unprocessed meat.

The “Traditional sedentary” pattern is also characterized by a more frequent consumption of refined grains, while loading negatively on whole grains. These characteristic point towards a high glycemic index (GI) dietary pattern. Even though the evidence on the association between GI and BP is conflicting, it is suggested that high GI diets lead to postprandial hyperglycemia, which in turn increases reactive oxygen species and lowers antioxidant concentrations. These changes are associated with increased BP and reduced endothelium-dependent blood flow [63]. It is important to note that the “Traditional sedentary” pattern has also loaded on dairy products, a food group that has been associated with protective effects against hypertension [64], given its unique micronutrient composition (vitamin D, calcium, phosphorous and potassium), its rich array of bioactive lactotripeptides and its low sodium content [64]. However, several large prospective cohort studies reported benefits from consuming low-fat *versus* whole-fat dairy products [65,66]. Dairy fat is primarily saturated fat, including medium and longer chain fatty acids that are atherogenic, which may potentially counter the benefits of dairy consumption [64]. The STEPwise survey conducted in Qatar did not allow differentiation between whole versus low fat varieties of dairy products.

This study has also examined the socio-demographic determinants of the identified lifestyle patterns. Adherence to the “Healthy” pattern was found to be associated with a higher education level. In line with these findings, Steele *et al.* (2014) showed that, amongst Brazilian adults, the prudent lifestyle pattern was positively associated with the number of schooling years. Higher education levels may in fact be associated with higher nutrition knowledge, an essential precursor to healthy dietary and lifestyle habits [67]. In our study, adherence to the “Fast food & smoking” pattern was associated with a higher consumption of meals outside home, while older and married women were less likely to adhere to this pattern. Similarly, Steele *et al.* (2014) showed that adherence to what they termed the “risky pattern”, characterized by the consumption of fat-rich meat, excessive alcohol and current smoking habits, was also found to be inversely associated with age. A possible explanation may be that older subjects tend to maintain traditional dietary and lifestyle habits as compared to younger generations who have greater exposure to “fashionable” foods and are more vulnerable to emerging marketing trends [68]. The inverse association between age and adherence to the “Fast food & smoking” pattern might also reflect a state of nutrition transition, from a “traditional” to a “western” lifestyle pattern, a phenomenon that typically manifests itself in younger age groups as is currently experienced by many countries of the Eastern Mediterranean region [69,70]. As for adherence to the “Traditional sedentary” pattern, it was found to be positively associated with parental consanguinity, which suggests that this pattern may be the closest to the traditional Qatari lifestyle pattern.

The present study had several strengths. Using the standardized WHO STEPwise survey approach in data collection and analysis, this study is the first to report on the association between lifestyle patterns and the risk of elevated BP. Its findings are stemming from a nationally representative sample of women of reproductive age, and weighting was performed to correct for the sample distribution. Interviewer errors and inter-observer measurement error in anthropometric assessment and BP measurement were minimized by extensive training of all interviewers to maintain quality of measurements and data collection The Arabic Global Physical Activity Questionnaire (GPAC) was used to assess physical activity, and its scoring followed a standardized approach, as recommended by the WHO. The derivation of lifestyle patterns provides a broader picture of food consumption and lifestyle components as they relate to disease risk, and may thus have more practical applications than the analysis of single foods, nutrients or behaviors.

Findings of this study should, however, be considered in light of the following limitations. First, the study had a cross-sectional design, and thus its findings can mainly be used to infer associations rather than assessing causal relationships. However, to eliminate possible reverse causation, we have excluded participants who reported having hypertension or those who reported dietary changes at the time of BP examinations (including patients with Diabetes). The rationale for the exclusion of participants with known diagnosis of hypertension was based on the fact that these participants might have received lifestyle consultations and have consequently changed their diet, exercise and smoking habits. These changes may dilute the effect of diet on elevated blood pressure and lead to a type II error.

Second, information on portion size was not collected. However, it is important to note that the complex cognitive process of portion size estimation may pose additional challenges to study participants who consume varying portion sizes across meals [71] and may not be always aware of the portion size [72]. Although quantification skills may improve with training and the use of food photograph aids [73], inclusion of portion size questions may increase respondent burden and lead to data omission, and hence contribute only marginally beyond frequency data in improving validity of the dietary assessment tool [74]. Therefore, recent research in this area has focused on the development of non-quantitative dietary questionnaires (without collection of portion size information) as targeted dietary assessment tools to rank individuals by intake of specific food groups or dietary patterns rather than providing absolute values for foods and/or nutrients [75]. Besides providing information on usual intakes of a particular food or food groups of interest, such questionnaires are particularly useful in identifying dietary patterns at the population level [76]. Third, salt intake, which is an important factor influencing BP, was not assessed in the present study. Most studies investigating the association between dietary patterns and BP did not examine salt intake, since valid assessment of salt intake necessitates the collection of 24-hour urine samples or the adoption of robust dietary assessment tools [77]. Lastly, it remains important to note that no information on alcohol intake was obtained, the latter being a possible risk factor for elevated BP. For culture-specific reasons, this information was not collected in the Qatar STEPwise survey.

## 5. Conclusions

This study documented a significant positive association between elevated BP and specific Qatari lifestyle patterns amongst women of reproductive age. More specifically, the “Fast food & smoking” pattern (smoking, and consumption of fast foods, sweetened beverages, and sweets) and the “Traditional sedentary” pattern (physical inactivity and the consumption of meat and refined grains) were associated with an approximately two-fold increase in the risk of elevated BP in this age group. These findings highlight the synergistic effects that diet and other lifestyle components may have on disease risk.

The results of this study therefore support the recommendations of the Eighth Report of the Joint National Committee on Prevention, Detection, Evaluation, and Treatment of High Blood Pressure (JNC 8) and the European Society of Hypertension/European Society of Hypertension of Cardiology, which state that appropriate lifestyle interventions, including increased physical activity and the adoption of healthy dietary patterns, should be fostered for the prevention and treatment of hypertension [51]. Qatar launched, in April 2015, the Qatar Dietary Guidelines, which aim at directing both individual behavior change and the development of health and food policies in Qatar [78]. It would be of value to revisit the guidelines in light of the information provided in this study and investigate the impact of these guidelines on dietary habits of the population in general and of Qatari women in particular.
